# Microbial diversity and production of milk spirit using traditional Buryat fermentation and distillation technologies

**DOI:** 10.1038/s41598-026-45709-5

**Published:** 2026-04-16

**Authors:** Zorigto Namsaraev, Bair Nanzatov, Aleksandra Kozlova, Darima Barkhutova, Anna Izotova, Anna Vlaskina, Dmitry Petrenko, Andrey Kamaev, Svetlana Lukashevich, Viktor Pozhidaev, Alla Filimonova, Dulma Tsyrenova, Vyacheslav Dambaev, Valeriia Novikova, Aleksei Rozanov, Alexey Sazonov, Maksim Patrushev, Stepan Toshchakov

**Affiliations:** 1 NRC “Kurchatov Institute”, Moscow, Russia; 2 Sirius University of Science and Technology, Sirius Federal Territory, Sirius, Russia; 3 Moscow Center for Advanced Studies, Moscow, Russia; 4 Institute for Mongolian, Buddhist and Tibetan Studies, Siberian Branch of the Russian Academy of Sciences, Ulan-Ude, Russia; 5 International Laboratory of Bioinformatics, HSE University, Moscow, Russia; 6 Center for Molecular and Cellular Biology, Moscow, Russia; 7 Institute of General and Experimental Biology, Siberian Branch of the Russian Academy of Sciences, Ulan-Ude, Russia

**Keywords:** Milk fermentation, Alcoholic distillates, Khurenge, Darasun, Buryat, Mongolic peoples, Biotechnology, Microbiology

## Abstract

Distilled fermented milk beverages are rare in food technology, despite the global prevalence of plant-based spirits. Currently, the production of distilled strong alcoholic beverages from fermented milk using traditional technologies is known only among Mongolic-speaking peoples and their Siberian neighbors. This study provides the first interdisciplinary analysis of *darasun*, a traditional Buryat spirit made from fermented milk beverage *khurenge* in Southern Siberia. Archival research shows deep historical roots of *darasun* dating back to the 13th–14th centuries in the period of the Mongol Empire. Low content of fermentable carbohydrates in milk limits the maximum ethanol concentration in the fermented product, which reaches 1.5–2.1%. To increase the beverage’s strength, a traditional multi-stage distillation is used, allowing sequential increases in ethanol concentration to 11%, 40%, and 75%. ITS and 16S rRNA gene sequence analysis identified diverse microbial community in *khurenge* dominated by representatives of *Lactobacillus* genus among lactic acid bacteria and *Trichosporon or Monosporozyma* genera among yeasts. Comparison of home-produced and industrially produced *khurenge* revealed that pasteurization significantly affects microbial composition with non-pasteurized *khurenge* showing greater microbial diversity and lower ethanol content than pasteurized. Functional analysis showed that pathway composition differed significantly between samples suggesting that final ethanol concentration in fermented milk reflects the balance between competing fermentation and respiration pathways. This study demonstrates that Buryat milk fermentation and distillation represents a unique biotechnological system that exemplifies decentralized, low-input, circular bioeconomy principles adapted to pastoral settings, providing a contrast to industrial whey-to-ethanol processes and contributing to the preservation of endangered cultural heritage.

**Supplementary Information:**

The online version contains supplementary material available at 10.1038/s41598-026-45709-5.

## Introduction

The development of distilled alcoholic beverages marked a significant technological milestone in human civilization, emerging considerably later than fermented beverages such as beer and wine^1–3^. According to current evidence, the earliest known findings of possible distillation apparatuses originate from Mesopotamia and China and date to between 3500 and 2000 B.C.E.^4^. In written sources, the earliest references to the concept of distillation, though not specifically for potable alcohol, are associated with Aristotle (350 B.C.E.), Maria the Jewess (1st century C.E.), and Jabir ibn Hayyan (Geber) (8th -9th centuries C.E.)^5,6^. By the Middle Ages, the technology for producing alcoholic distillates had already spread widely across various parts of Eurasia, including China, Mongolia, India, the Middle East, and Europe^7–11^.

**Fig. 1:**
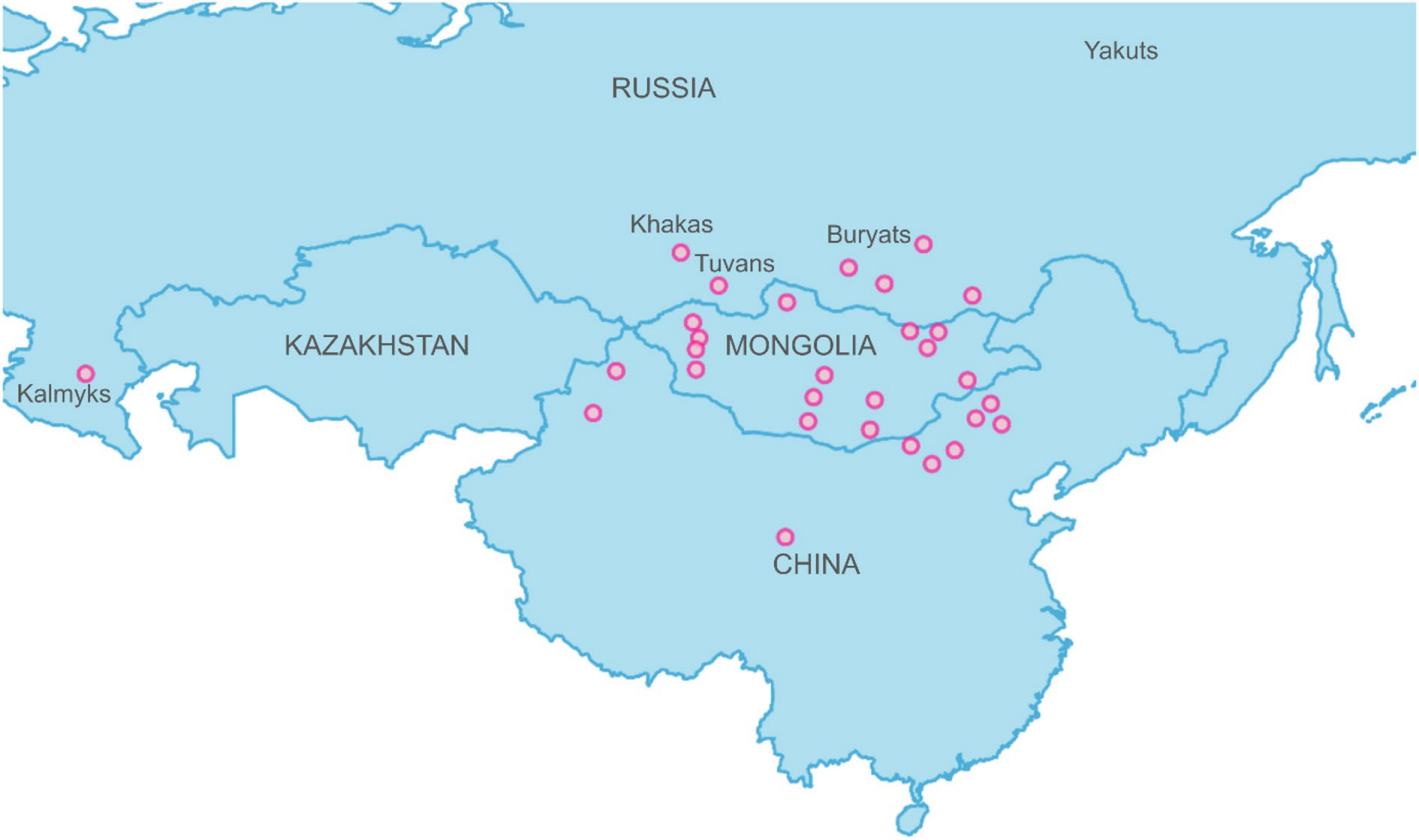
Map illustrating the distribution of traditional technology for distilling alcohol from fermented milk across Eurasia. The locations of points in Mongolia and China are based on data from works of Hirata^12,13^.

The overwhelming majority of distilled products are derived from fermented plant-based materials, whereas the distillation of fermented milk is extremely rare^12,13^. In the nomadic and pastoral cultures of Eurasia, low-alcoholic milk beverages such as kumis, kefir, shubat, ayran, and others are widespread^14–16^. Nevertheless, the distillation of fermented milk to produce strong alcoholic beverages is found only among Mongolic-speaking peoples (including Buryats and Kalmyks) and their geographically proximate neighbors, the Tuvans and Khakas (both Turkic-speaking) in southern Siberia, but not among the Yakuts and Kazakhs^12,13,17–21^(Fig. 1).

Research on mildly alcoholic fermented dairy products of Mongolic peoples is well represented in the scientific literature. Studies have shown that these beverages are dominated by lactic acid bacteria, including *L. delbrueckii*,* L. helveticus*,* L. kefiranofaciens*, and *Streptococcus thermophilus*, alongside representatives of novel species such as *Bifidobacterium mongoliense*^22–30^. Newly isolated strains from these products may possess beneficial properties, including antibacterial and proteolytic activity, and show promise for treating dysbiosis in children^31,32^. The production of stronger beverages from milk via distillation has been discussed in articles focusing on the material culture and traditions of Mongolic peoples^12,19,33^. However, existing publications do not address why the tradition of producing strong alcoholic beverages from fermented milk persists only among Mongolic-speaking peoples and their immediate neighbors, nor whether it is linked to specific technological approaches or the distinctive characteristics of starter culture microbial communities.

The traditional milk fermentation and distillation technology maintained among Buryat populations represents one of the few extant examples of ethanol production from milk. Buryats, a Mongolic-speaking people inhabiting southern Siberia, Mongolia, and China, have preserved a tradition of distilling alcohol from fermented milk, which is produced using backslopping starter cultures maintained within family units. We conducted the first study of the microbial diversity of the starter culture *khurenge* and Buryat traditional technology complex for producing the alcoholic distillate *darasun* from the fermented milk. Based on our findings, we hypothesize that the development and preservation of the traditional complex of milk distillation technologies among Mongolic peoples is linked to the lack of stable access to substrates rich in fermentable carbohydrates. This limitation was compensated for by repeated distillation of the alcohol-containing product obtained.

## Results

### Traditional milk processing system in Buryat Republic

**Fig. 2:**
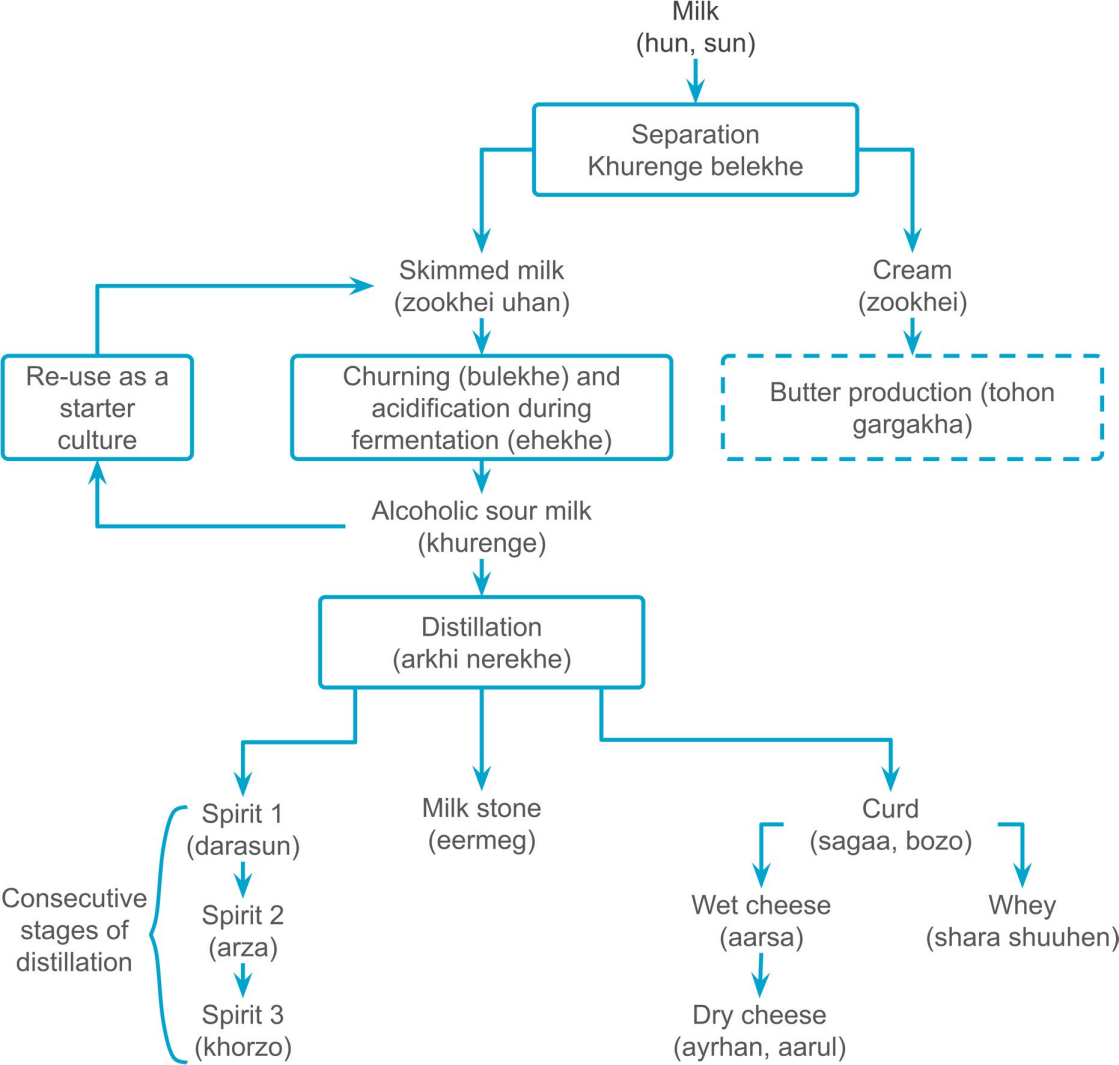
Milk processing system illustrating its fermentation and distillation with by-product recovery in Buryat Republic.

Currently, the whole milk (*hun / sun*) is typically separated using an electric separator to obtain cream (*zookhei*), which is further processed to make butter (*tohon / toson*) (Fig. 2). The resulting skim milk is used either for making milk tea (*hutei sai / sutei tsai*) or for producing fermented dairy beverages (*khurenge / airag*). Before fermentation, the milk may be pasteurized. However, in home production, unpasteurized milk is generally used.

In contemporary home production, the following proportions and fermentation vessel sizes are commonly employed: 40–50 L of milk are poured into a 70-liter plastic barrel, which contains 10–15 L of khurenge from the previous fermentation cycle (*khurengen ekhe*, Buryat: “mother khurenge”). The barrel is sealed with a plastic lid and kept at ambient temperature in outbuildings. According to local observations, the optimum temperature for preparing khurenge is approximately 20 °C. The fermentation cycle lasts 2–3 days. During fermentation, carbon dioxide is produced in the milk, causing foam to rise to the surface. Beneath the foam, a layer of whey forms, while a layer of coagulated protein accumulates at the bottom. The khurenge is actively stirred (*bulekhe*) 2–3 times a day to break up the foam, which also promotes acidification (*ehekhe*) of the beverage.

Khurenge is classified into three stages of maturation: weak, medium, and strong. Weak khurenge matures within 24 h, has a pleasant sour-alcoholic taste, Turner acidity of 100–120°, and an alcohol content of approximately 0.2%. Medium khurenge matures over two days, displays a pronounced sour–alcoholic flavor, Turner acidity of 160–200°, and contains 0.3–0.6% alcohol. Strong khurenge matures after three days, is characterized by a bitter taste, Turner acidity of 240–300°, and an alcohol content of 0.6–1.2%^34^. Despite the simplicity of the technology, defects may occur during khurenge preparation. One known defect is termed *arabka*. In this condition, khurenge exhibits a pronounced bitter taste, unpleasant odor, lacks foam formation, has a top layer consisting solely of whey, and all casein flakes settle in the bottom layer^34^. The occurrence of this defect may be associated with the activity of proteolytic microorganisms and low levels of lactic acid fermentation.

After the gas production process subsides, likely due to decreased fermentation activity, the milk is transferred to a vessel for distillation. Typically, household distillation of khurenge is performed without temperature control, yielding approximately 1 L of darasun with an alcohol content of around 10–11% from 10 L of khurenge. The resulting product is bottled in plastic or glass bottles and is suitable for long-term storage.

Considering the lower content of fermentable sugars in milk compared to plant substrates, the preparation of alcoholic beverages from milk requires a significantly larger volume of raw material. In the early 20th century, according to ethnographic diary notes by Ts. Jamtsarano recorded in 1906 in the ulus of Olzony, Verkholeksky district, Irkutsk Province, the Buryats were compelled to perform multiple distillation cycles, accumulating and blending the distilled products: “they distilled in advance and in large quantities, typically up to 40 times”^35^. The resulting mixture would be referred to as darasun of a certain number of cups (cauldrons), for example, 40 cups. It was believed that the stronger the mixture, the more pleasing it was to the deities—“arak is sprinkled from three, five, eight distillations, sometimes even forty”, “araka was of nine distillations (yuhen togoonoi)”^35^.

If desired, darasun can undergo additional distillation steps to increase the alcohol strength. In 20th-century Mongolia, N. L. Zhukovskaya documented five stages of distillation: *arkhi*,* arza*,* khorzo*,* sharza*, and *dun*, with alcohol content rising from approximately 9–11% to 50%^36^. Using traditional equipment with low sealing efficiency likely required many distillation stages, whereas apparatuses that are more modern achieve high alcohol concentrations within 2–3 stages. For example, in Buryatia during 1941–1942, Filippov reported three distillation stages yielding *arkhi* (12–15% alcohol), *arza* (30–40%), and *khorzo* (50–70%)^34^. Our experimental results corroborate Filippov’s data: during experimental distillation of darasun containing 11.2% ethanol using a laboratory distillation flask and Liebig condenser, the *arza* fraction (second distillation) contained 40–45% ethanol, while the *khorzo* fraction (third distillation) reached 75–77% ethanol.

During the distillation of darasun, various by-products are formed, many of which have traditional uses^37^. After distillation, a residue known as milk stone (*eermeg*) remains on the walls of the still and can be consumed as food. The dense milk mass left in the still after distillation is called *sagaa* or *bozo*. This mass is poured into a special vessel (*torkho*) with holes at the bottom, allowing the whey (*haib*,* shara shuuhen*) to drain off. Alternatively, the mass may be strained through a special bag (*shuumeg*). A thick curd accumulates in the vessel or bag, referred to as *aarsa*. *Aarsa* is dried to produce a hard product called *airhan* or *aruul*, which is suitable for long-term storage.

### Historical and etymological analysis of the terms khurenge and darasun

In the Buryat traditional system of obtaining alcohol distillates from milk, there are two main terms - khurenge and darasun. In different ethno-territorial groups of Buryats, there is terminological diversity in these terms. The synonyms *kyurenge*,* köröngö*,* körüngge*,* airag* can be used for the khurenge. Whereas *tarasun*,* hurengyn arkhi*,* kurengyn arsi*,* togonoi arkhi*,* shimiin arkhi* could be used as synonyms for darasun.

The authors of the etymological dictionary of Mongolian languages, regarding the term *körüngge* – ‘starter culture’, assume its Mongolian origin, as well as borrowing from Mongolian into the Kyrgyz language: *köröŋgö* and into the Yakut language: *köyörö*. Moreover, the Yakut form probably reflects the process of transition from *könerge > *köyörgö*, and the known Mongolian forms reflect the metathesis *könerge > *körenge > *körüngge^38^. One of the earliest mentions of the term *körüngge* in written sources appears in the Mongolian law code “Khalka Jirum,” revised in 1709. Section  33(6) refers to a penalty for the theft of starter culture, “basa körünge-tü ayiraγ”, which amounted to two head of large livestock and three sheep^39,40^. Considering the severity of the fine and the inclusion of starter culture in the legal code, khurenge represented a commodity of significant value in the pastoralist economy.

In modern Mongolic languages, the term darasun was recorded in Khalkha-Mongolian as *dars* – ‘fruit wine’; in Kalmyk *darsn* with the meanings ‘fruit wine’ or ‘bread vodka’; in Dagur *darsa*; in Dunxiang *darasun*; in Monguor *derāse*, in addition to the first two meanings, ‘juniper tincture’ is added; in Buryat it is found in the meaning ‘milk vodka’^41^. Originally, the term darasun was recorded in Mongolic during the era of the Mongolian Empire. One of the earliest precise mentions (14th century) of the term darasun was recorded in the glossary of Jamal ad-Din Ibn Muhanna as darāsūn meaning ‘wine’^42^. Another piece of material evidence for the term darasun is a vessel in the British Museum, number 1927,0217.1, dating from the Yuan Dynasty (1260–1368) (https://www.britishmuseum.org/collection/object/A_1927-0217-1). S. Jenyns, who published a description of this vessel, asked Professor P. Pelliot for help in deciphering the inscription, and recorded that the vessel bears an inscription in Mongolian script (Phags-pa), as *sayi darasun jy* – ‘good wine’ ^43^. It should be noted that the term *jy* probably reflects the Chinese term 酒 *jiǔ* meaning ‘alcoholic [drink]’, which goes back to the etymological meaning of ‘something intoxicating’, since this character, for example, is included in the concept of kumiss - 馬奶酒 *mǎ nǎi jiǔ* – literally ‘wine from mare’s milk’.

An indirect piece of evidence is the mention of the term ‘terracinam,’ meaning ‘rice beer,’ made by the Franciscan monk Guillaume de Rubruck. In 1254, he visited Mongke Khan of the mongols in his capital Karakorum and left a description of his palace (https://www.digitale-sammlungen.de/en/view/bsb10901329?page=239). In particular, he described a fountain from which four types of drinks flowed: “great silver tree, and at its roots are four lions of silver, each with a conduit through it, and all belching forth white milk of mares. And four conduits are led inside the tree to its tops, which are bent downward, and on each of these is also a gilded serpent, whose tail twines round the tree. And from one of these pipes flows wine, from another caracosmos, or clarified mare’s milk, from another bal, a drink made with honey, and from another rice mead, which is called *terracina*; and for each liquor there is a special silver bowl at the foot of the tree to receive it. Between these four conduits in the top, he made an angel holding a trumpet, and underneath the tree he made a vault in which a man can be hid. And pipes go up through the heart of the tree to the angel. In the first place he made bellows, but they did not give enough wind. Outside the palace is a cellar in which the liquors are stored, and there are servants all ready to pour them out when they hear the angel trumpeting. And there are branches of silver on the tree, and leaves and fruit. When then drink is wanted, the head butler cries to the angel to blow his trumpet. Then he who is concealed in the vault, hearing this blows with all his might in the pipe leading to the angel, and the angel places the trumpet to his mouth, and blows the trumpet right loudly. Then the servants who are in the cellar, hearing this, pour the different liquors into the proper conduits, and the conduits lead them down into the bowls prepared for that, and then the butlers draw it and carry it to the palace to the men and women”^44^.

For the purpose of this study, the most important part of the text is the mention of four beverages, which in the original Latin text appears as follows: “… ipse fecit a nobis queri quod vellomus bibere utruni vinum vel *terracinam*, hoc est cervisium de risio, vel caracosmos, hoc est darum lac iumenti, vel bal, hoc est medonem de melle.”^45^. It is noteworthy that in the translation of Rubruck by W.W. Rockhill into English, the term *terracinam* was already compared with *tarassun*^44^. In the translation into German, published in 1934, the term *terracinam* was also compared with *tarasun*: “… das auch *tercina (tarasun)* hei$\mathcal{B}$t” ^46^. Subsequently, L.W. Clark, examining the Turkic and Mongolic vocabulary in Rubruck’s work, supported Rockhill in comparing *terracinam* with the Mongolian darasun, where he also questioned the influence of the Persian *darčin* meaning ‘cinnamon’ on the Latin distortion^47^.

The form *tarasun*, widespread in the Russian dialects of the Baikal region, reflects a borrowing from the Buryat language, which was widespread among the Russian population. In Russian documents of the 18th century, the term, along with others, was actively used in office work^48^. The Russian form *tarasun* reflects a borrowing from the Buryat language before the transition s > h, namely *darasun > darahun > darahan*, i.e. at least by the 1720s, which reflects the existence of this word in the Buryat language at least in the 17th century^49^. Probably the earliest evidence of the use of the term *tarasun* by Russian officials is its recording by J.G. Gmelin in 1736, during his stay in Bratsk Fort, in a story about the events associated with the destruction of a Russian squad by Buryats in 1652. The text mentions that the Buryats “gave the guests tarasun or kumys to drink”^50^. In the mid-18th century, active cross-border trade of darasun between China and Russia existed in Siberia^51^. In the ethnographic works of M.N. Khangalov, collected by him in the second half of the 19th century, *tarasun* is mentioned repeatedly^52^. 

In addition, a widespread term reflecting the concept of sour milk alcoholic distillate in the Buryat language is *togoonoi arkhi* or *togooni arkhi*. Currently, the distorted form *togone arkhi* is also found. The term is based on *toγuγan > toγoon* – ‘cauldron’^53^ and *araki > arkhi* – ‘vodka’^41^. Regarding the term *araki*, there is an established opinion about its Arabic origin from *ariqa* and its wide penetration into the Altaic languages, as well as into the languages of their neighbors through the Turkic and Mongolian languages, however, the authors of the “Etymological Dictionary of Mongolian Languages” consider this position controversial^41^. Another name for sour milk alcoholic distillate is the term *khurengyn arsi / arkhi*,* kürengyn arsi / arki*, associated with a specific stage of production of this drink from khurenge^37^.

### Description of the khurenge samples

Khurenge samples HA, produced in a household setting, contained an average of 1.5% (v/v) ethanol, had a pH of 3.3 and a titratable acidity of 240 Turner degrees. Khurenge samples HU contained 2.1% (v/v) ethanol, exhibited a pH of 3.7 and titratable acidity of 160 Turner degrees. Both liquids were white in color and showed vigorous gas formation. The aroma is sour with fermented dairy notes. The taste is strongly acidic and sour with pronounced milk character. The mouthfeel and texture are thick, viscous, and creamy, with a slight fizzy sensation from residual CO₂ produced during fermentation. The aftertaste is acidic and refreshing. Sample HA displayed a more pronounced acidic flavor profile than sample HU. The darasun sample produced from HA khurenge contained ethanol at a concentration of 11.2% (v/v). The main organoleptic characteristics of the produced darasun include milky and cheese-like tones, alongside subtle animal notes, hints of leather, and dry herbs. The aftertaste is acidic and refreshing. The color is clear with a slight whitish turbidity.

The carbohydrate and organic acid profiles of khurenge are consistent with lactose fermentation resulting in the production of lactic acid, ethanol, glucose, galactose, and other metabolites. In HA khurenge, analysis by GC–MS of silylated derivatives revealed a range of carbohydrates, dominated by lactose isomers. Smaller amounts of D-glucose and D-galactopyranose (a tautomeric form of D-galactose) were also detected (Table 1). A number of organic acids were present, with lactic acid being the dominant component; 2-hydroxy-3-methylbutyric acid, 2-hydroxyisocaproic acid and succinic acid were also detected at concentrations exceeding 1% (Table 2). In HU khurenge, the carbohydrate composition was generally similar to that of HA, whereas differences were observed in organic acid composition. In addition to 2-hydroxy-3-methylbutyric acid, 2-hydroxyisocaproic acid and succinic acid, HU samples also contained acetin (glycerol esters of acetic acid) and 2-hydroxy-3-methylvaleric acid. The acetic acid content, determined by HPLC, was negligible (< 1%) in both khurenge types.

**Table 1:** Comparative carbohydrate content in khurenge samples.

Carbohydrate	HA	HU
	Rel. conc.*	Rel. conc.
d-Glucose	3.9	2.7
d-Galactopyranose	7.2	5.1
d-Lactose, (isomer 1)	100	100
d-Lactose, (isomer 2)	71.5	36.56

Rel. conc. * - The relative concentration of each component was expressed relative to the peak with maximum area. The table presents data for compounds with relative concentrations above 1%.

**Table 2:** Comparative organic acids content in khurenge samples.

Organic acid	HA	HU
	Rel. conc.*	Rel. conc.
Lactic acid	100	100
2-Hydroxy-3-methylbutyric acid	5.9	2.9
Acetin	–	9.9
2-Hydroxyisocaproic acid	6.6	4.4
2-Hydroxy-3-methylvaleric acid	–	2.2
Succinic acid	4.4	2.0

Rel. conc. * - The relative concentration of each component was expressed relative to the peak with maximum area. The table presents data for compounds with relative concentrations above 1%.

**Fig. 3:**
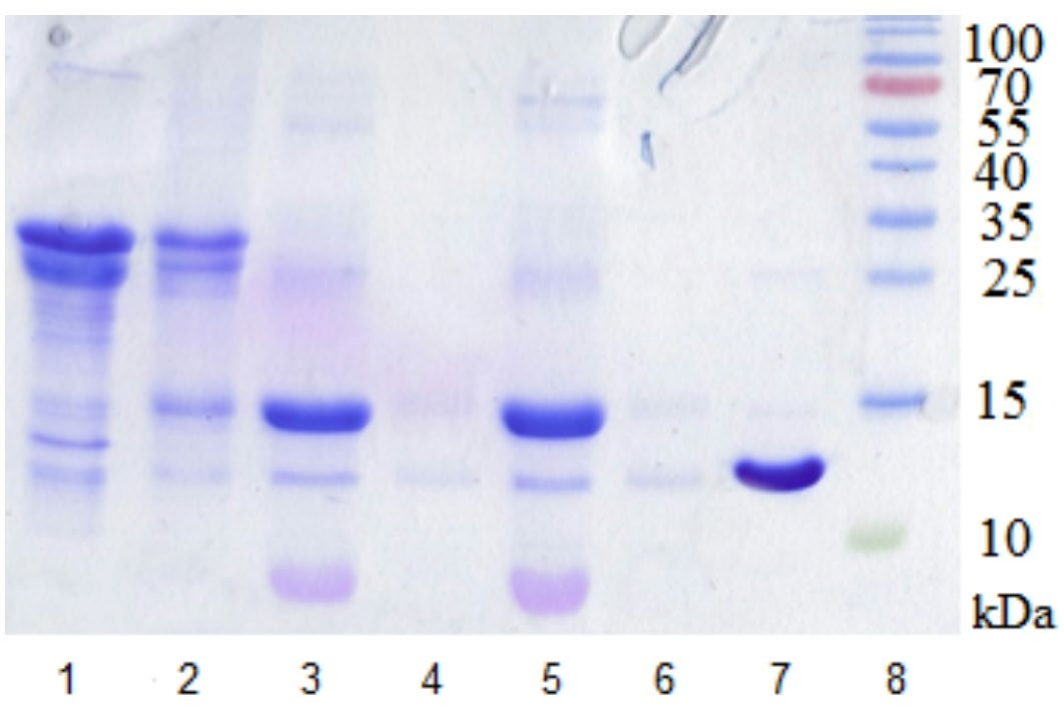
Protein electrophoresis of khurenge samples. Lanes from left to right: (1) Precipitate of home-produced khurenge HA, (2) precipitate of second khurenge HU, (3) whey of HA with pH 3.6, (4) whey of HU with pH 3.6, (5) neutralized whey of HA pH 7.0, (6) neutralized whey of HU pH 7.0, (7) lysozyme (M = 14 kDa), (8) molecular weight marker.

Protein content in khurenge samples was measured in whole beverages and whey obtained by centrifugation. HA contained 1.35% total protein and 0.47% in whey, while HU had 0.93% total protein and 0.17% in whey. Caseins (25–35 kDa) predominated in precipitates of both samples. A key difference was the presence of β-lactoglobulin (15–18 kDa) in HA whey but as part of the precipitate in HU whey, indicating that HU milk underwent heat treatment causing β-lactoglobulin coagulation (Fig. 3). Both whey fractions contained α-lactalbumin (~ 14 kDa), which remained soluble after heating^54^. Experimental heating and fermentation trials confirmed that HU was produced from thermally processed milk, whereas HA was unheated, consistent with household preparation.

### Microbial community composition of khurenge

**Fig. 4:**
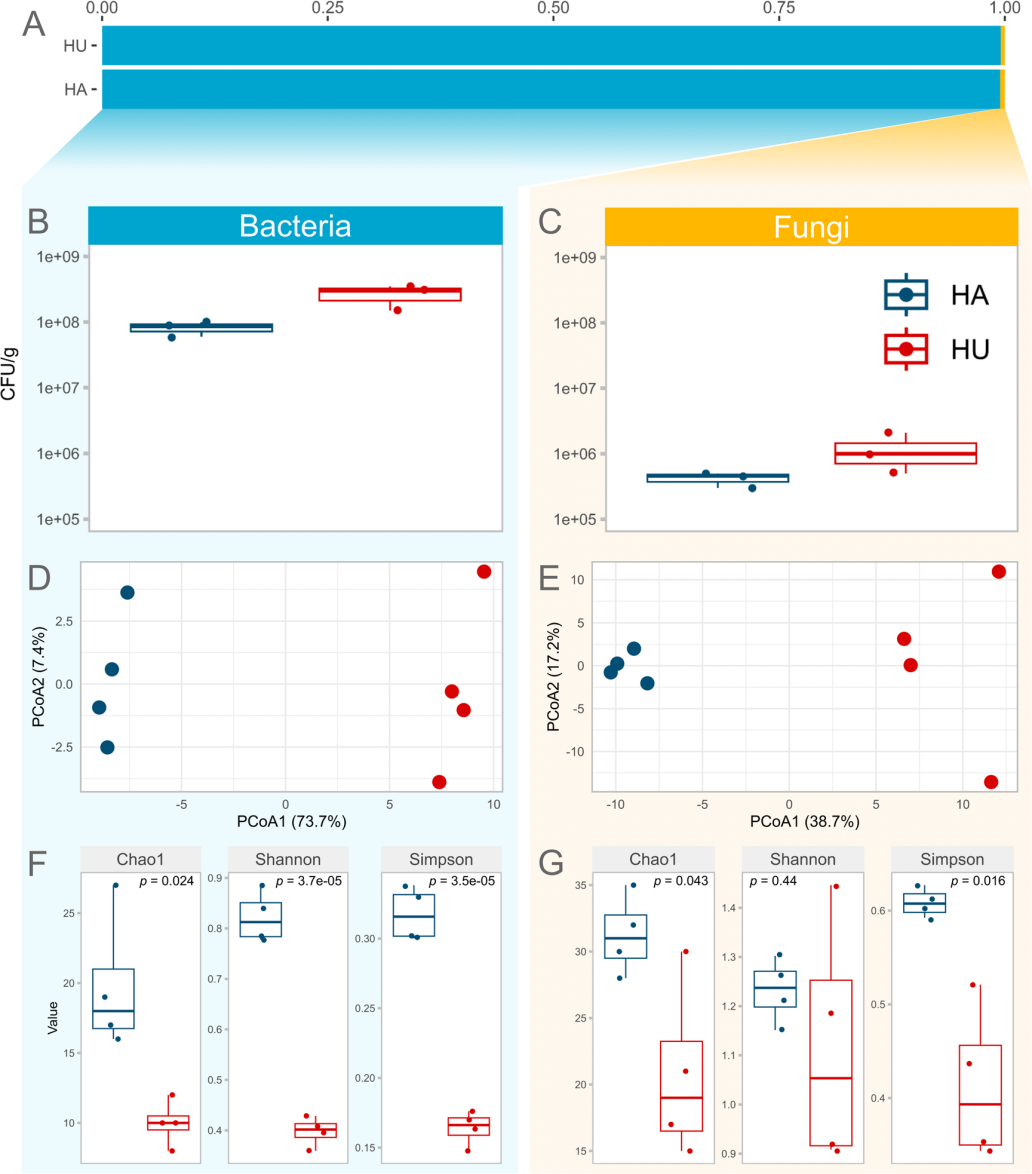
Comparison of HU and HA for separated kingdoms of Bacteria and Fungi. (**A**) Relative abundance of kingdoms in the HA and HU samples based on CFU/g data. Blue represents the bacterial kingdom, and orange represents the fungal kingdom. (**B**, **C**) Differences in CFU/g between HU and HA samples for the bacterial and fungal kingdoms, respectively (log scale). (**D**) Beta-diversity based on Aitchison distance with PCoA visualization at the species level for the bacterial kingdom only. (**E**) Alpha-diversity calculated using Chao1, Shannon, and Simpson indices for the bacterial kingdom only. Comparisons were performed using t-tests with adjusted p-values (p values shown on the plot). (**F**) Beta-diversity based on Aitchison distance with PCoA visualization at the species level for the fungal kingdom only. (**G**) Alpha-diversity calculated using Chao1, Shannon, and Simpson indices for the fungal kingdom only.

Microbial enumeration analysis showed that in all khurenge samples the bacterial population exceeded the yeast population by two orders of magnitude. In HA lactic acid bacteria accounted for 8.2 × 10^7^ CFU/g and yeasts for 4.2 × 10^5^ CFU/g. In HU lactic acid bacteria numbered 2.7 × 10^8^ CFU/g and yeasts 1.2 × 10^6^ CFU/g (Fig. 4).

The microbial communities of khurenge samples were analyzed by high-throughput sequencing of the V4 hypervariable region (16 S rRNA gene) and the ITS amplicons. In total, four samples (two samples from each khurenge type) were analyzed, each in two independent PCR replicates. After primary quality control, including read filtering, merging of overlapping read pairs, and removal of chimeric sequences, approximately 7,700 reads for bacteria and 13,512 reads for fungi were retained for further analysis. Rarefaction analysis, performed with the vegan package, showed that all samples were sequenced with a sufficient depth (Supplementary Material 1, Figs. 1 and 2).

### Beta-diversity

We first tested whether microbial diversity differed between the two groups of samples, separately for bacteria and for fungi. For the bacterial kingdom, multivariate homogeneity of group variances based on Aitchison distances was assessed using the betadisper function of the vegan package. Variances in the two clusters were homogeneous (*p* > 0.05), justifying the use of permutational multivariate analysis of variance (PERMANOVA) to test for differences in microbial community composition between these groups. PERMANOVA, performed using the adonis function, revealed statistically significant differences between HA and HU samples (*p* = 0.025), with the khurenge type explaining 73.2% of the variance in the prokaryotic microbiome (*R²* = 0.732). Principal coordinates analysis (PCoA) ordination also showed clear clustering of HA and HU samples, with distinct separation of centroids between groups (Fig. 4).

The same analysis was applied to the fungal kingdom. Multivariate homogeneity of group variances based on Aitchison distances, assessed with betadisper, indicated that variances were also homogeneous (*p* > 0.05). This again justified the use of PERMANOVA, which showed statistically significant differences between HA and HU samples (*p* = 0.033), with the khurenge type explaining 37.6% of the variance in the eukaryotic microbiome (R² = 0.376). PCoA ordination similarly showed clear clustering of HA and HU samples, with distinct centroids for each group (Fig. 4), consistent with the prokaryotic microbiome pattern. Having observed differences between the groups in β-diversity, we next assessed α-diversity to evaluate within-sample microbial richness and evenness.

### Alpha diversity

Amplicon sequence variants (ASVs) were determined using the DADA2 pipeline, which yielded 43 ASVs assigned to 12 bacterial genera (including “Others”). Individual samples contained 8–27 bacterial ASVs, depending on khurenge type. t-tests showed significant differences in α-diversity metrics for the bacterial kingdom (*p* < 0.01), with HA samples exhibiting greater diversity and a higher proportion of specific microorganisms than HU samples. On average, 19 HA-specific ASVs were detected versus 10 HU-specific ASVs (Fig. 4). The Shannon, Simpson and Chao1 indices were also significantly higher in HA samples than in HU (0.822 and 0.397 respectively for Shannon index; 0.317 and 0.164 respectively for Simpson index, 19.75 and 10 for Chao1) (Fig. 4).

For the fungal kingdom, 111 ASVs were identified, belonging to 16 genera and 39 species. In several cases, multiple ASVs belonged to the same species, suggesting the presence of different fungal strains. As with bacteria, HA samples were more taxonomically rich than HU samples based on Simpson and Chao1 indices (*p* < 0.05) (0.608 and 0.412 for Simpson and 31.25 and 20.75 for Chao1 indices), but the Shannon index revealed no statistically significant differences (1.231 for HA and 1.115 for HU). Given the observed α-diversity differences, we further examined microbial community composition to identify taxa contributing to these patterns, including their centered log-ratio (clr)-transformed abundances across groups.

### Prokaryote community composition

**Fig. 5:**
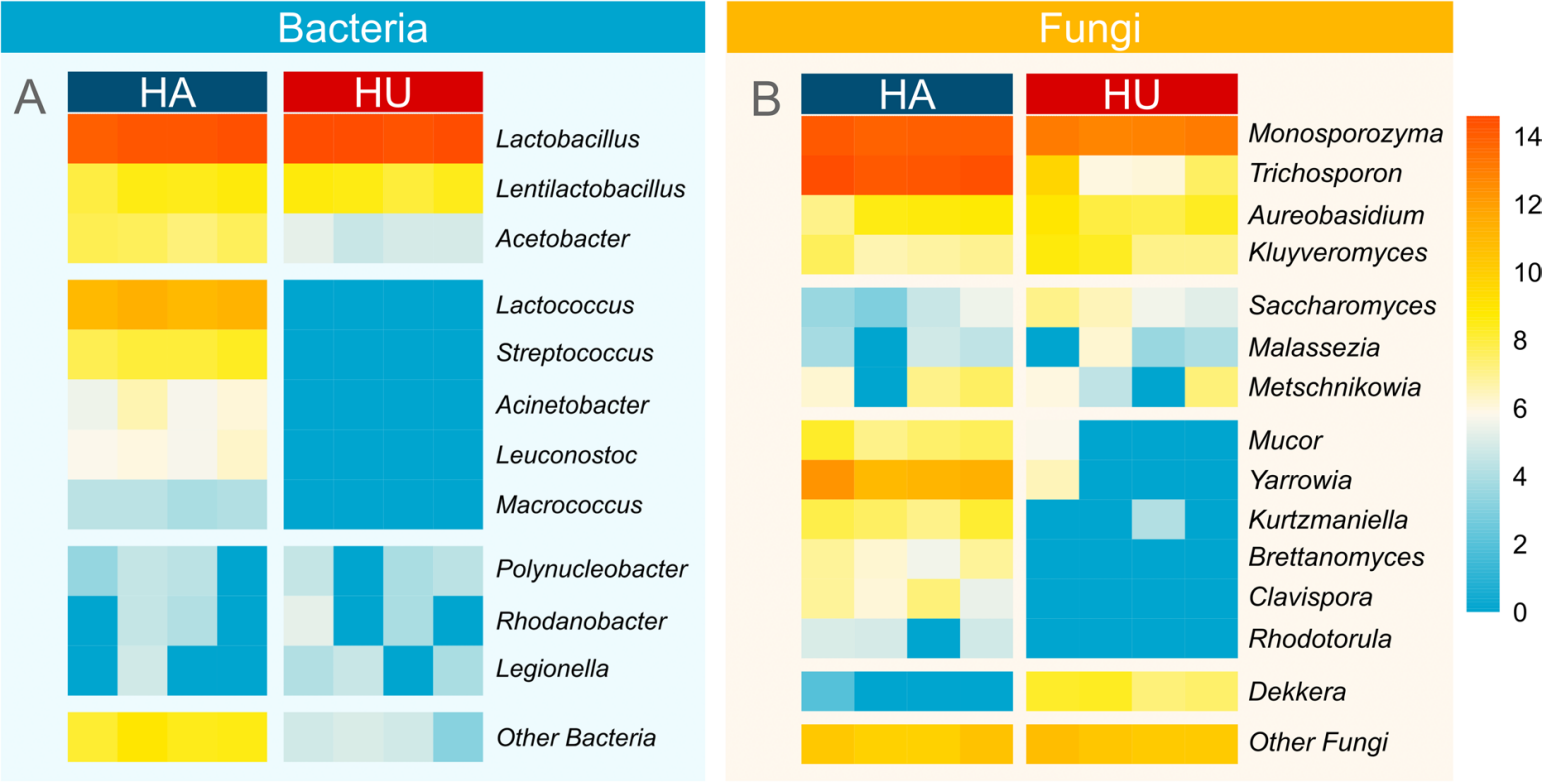
Heatmap of bacterial (**A**) and fungal (**B**) genera with centered log-ratio (log10) transformation in the HA and HU khurenge. Each column represents an individual sample including technical replicates. Clustering of rows was performed based on Euclidean distances. The class “other” was separated into a distinct group in both kingdom heatmaps. The scale on the right indicates the range of log-transformed counts.

The most abundant genome-equivalent taxon in both types of khurenge was the order *Lactobacillales* (up to 97% in HA and 99% in HU) (Fig. 5). Both HA and HU datasets were dominated by a genome-equivalent ASV assigned to genus *Lactobacillus*, comprising 80–83% of reads in HA and 90–92% in HU samples. BLAST analysis indicated its 100% sequence identity with *Lactobacillus helveticus*. Three additional ASVs from HU clustered with *L. helveticus* at 99.6% identity and accounted for 5–6% and < 1% of the prokaryotic community, respectively. In HA samples, in addition to the dominant ASV, representatives of *Lactococcus* (6–7%), identified by BLAST as *Lactococcus lactis* (3–4%), *Lentilactobacillus* sp. (1%), *Enterococcus* sp. (1%) and *Streptococcus* sp. (1%) were present. In HU samples, besides ASVs related to *L. helveticus*, *Lentilactobacillus* sp. (1%) was detected.

Among the minor prokaryotic groups (< 1% relative abundance), representatives of the genera *Lactobacillus* (further identified by BLAST as *Lactobacillus kefiranofaciens*, 99.6% sequence identity), *Polynucleobacter* and *Legionella*, as well as the species *Rhodanobacter thiooxydans* and *Aurantimicrobium minutum*, were shared between HA and HU samples. HA samples exclusively contained members of the families *Rhodocyclaceae*, *Neisseriaceae*, *Comamonadaceae* and *Babeliaceae*, as well as the genera *Macrococcus*, *Acinetobacter*, *Clostridium*, *Rothia* and *Catenisphaera*, and the species *Streptococcus dysgalactiae*. In contrast, HU samples exclusively harboured representatives of the family *Carnobacteriaceae* and the genera *Acetobacter*, *Massilia*, *Rurimicrobium* and *Alcaligenaceae* (GKS98 freshwater group), along with the species *Pseudarcobacter suis*.

### Fungal community composition

Comparative analysis of the fungal community composition revealed that HA and HU samples exhibited markedly different dominance patterns of the main species and significantly different diversity levels (Fig. 5). In HA samples, *Trichosporon coremiiforme* dominated (48–49%), followed by *Monosporozyma unispora* (formerly *Kazachstania unispora*) (35–39%). Together, these two species accounted for 84–89% of the total yeast community. Other taxa with > 1% relative abundance included *Yarrowia lipolytica* (5–10%), *Botryosphaeria* spp. (1%), and *Trichosporon* spp. (1%).

In HU samples, *M. unispora* was the dominant species (66–79%), whereas T. coremiiforme was present at only 1–6% relative abundance, similar to *Botryosphaeria spp.* (5–7%). Minor taxa (< 3%) included *Aureobasidium pullulans* (1–3%), *Kluyveromyces marxianus* (1–3%), *Dekkera anomala* (1–3%), *Saccharomyces paradoxus* (1%), *Metschnikowia reukaufii* (1%) and *Mycosphaerella sp.* (1%).

### Community metabolic prediction

**Fig. 6:**
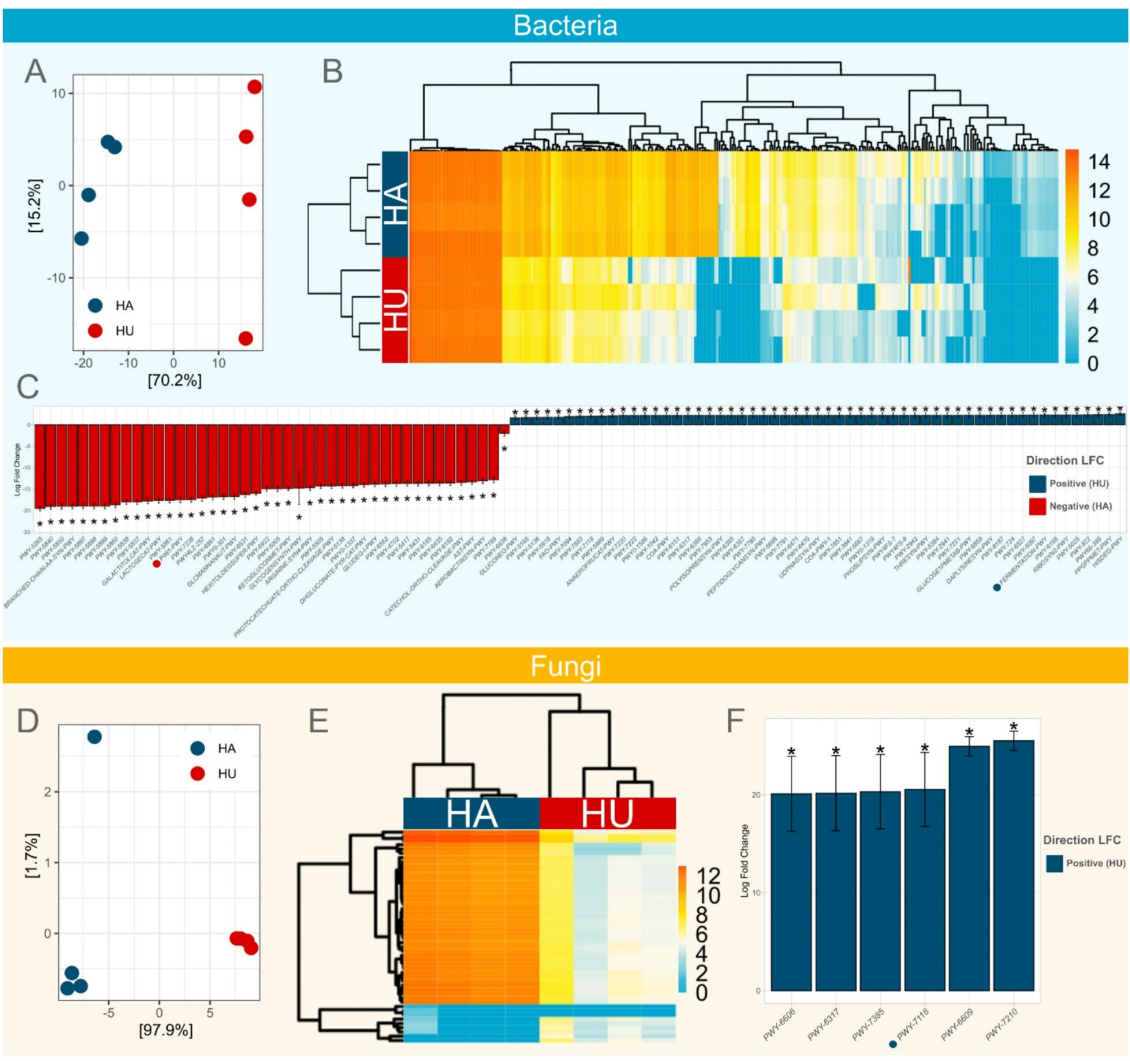
Comparison of predicted metabolic profiles between HU and HA samples. (**A**) Principal coordinate analysis (PCoA) based on Euclidean distances of predicted 16 S rRNA-derived pathway abundances, visualizing differences in metabolic profiles between samples. (**B**) Heatmap of predicted 16 S-derived metabolic pathways. Rows represent individual samples and columns represent predicted MetaCyc pathways. Colors indicate log₁₀-transformed relative abundance. Both samples and pathways were hierarchically clustered using Euclidean distance. (**C**) Differentially abundant predicted 16 S-derived metabolic pathways between HU and HA samples identified by ANCOM2. Each point represents a MetaCyc pathway, with the x-axis showing log fold change (log₂FC) in pathway abundance and the y-axis indicating the W-statistic from ANCOM2. Positive log₂FC values indicate higher relative abundance in HU samples; negative values indicate higher relative abundance in HA samples. (**D**) Principal coordinate analysis (PCoA) based on Euclidean distances of predicted ITS-derived pathway abundances, visualizing differences in metabolic profiles between samples. (**E**) Heatmap of predicted ITS-derived metabolic pathways. Rows represent predicted MetaCyc pathways and columns represent individual samples. Colors indicate log₁₀-transformed relative abundance. Both samples and pathways were hierarchically clustered using Euclidean distance. (**F**) Differentially abundant predicted ITS-derived metabolic pathways between HU and HA samples identified by ANCOM2. Each point represents a MetaCyc pathway, with the x-axis showing log fold change (log₂FC) in pathway abundance and the y-axis indicating the W-statistic from ANCOM2. Positive log₂FC values indicate higher relative abundance in HU samples; negative values indicate higher relative abundance in HA samples.

Using PICRUSt2, 1,473 ECs (Enzyme Commission numbers) and 294 metabolic pathways were predicted within the bacterial community component, with an average of 1,340 ECs and 273 metabolic pathways identified for HA samples and 1,133 ECs and 215 metabolic pathways identified for HU samples (Supplementary Material 2, Table 7, 8). In contrast, the fungal community component revealed approximately half as many ECs (692 total: 576 from HA and 604 from HU) and 50 metabolic pathways (42 pathways from HA and 47 from HU) (Supplementary Material 1, Table 9, 10). Multivariate dispersion of predicted microbial metabolic pathways did not differ between samples for either 16S- or ITS-derived profiles (betadisper, *p* > 0.05 for both). However, overall pathway composition differed significantly between beverages. Adonis2 revealed significant effects for both datasets: 16S-based pathways (*p* = 0.03, R² = 0.70) and ITS-based pathways (*p* = 0.027, R² = 0.96) (Fig. 6A, D).

To explore patterns in predicted metabolic functions, pathway abundances were visualized using a heatmap (pheatmap), with samples in rows and predicted pathways in columns. Both samples and pathways were hierarchically clustered based on Euclidean distances, identifying groups of samples with similar metabolic profiles and clusters of pathways exhibiting comparable abundance patterns (Fig. 6B). Several clusters were identified, including those containing pathways characteristic of both beverages. Pathways present in both HA and HU samples primarily included essential cellular life cycle pathways (PEPTIDOGLYCANSYN-PWY, PWY-2942, PWY-7791, etc.).

To identify metabolic pathways that differed significantly between HU and HA samples, ANCOM2 analysis was performed, identifying 101 discriminatory pathways (Fig. 6C). Particular attention was given to pathways involved in ethanol production and lactic acid fermentation. The hexitol fermentation pathway (P461-PWY), which produces lactate, formate, ethanol, and acetate, was significantly more abundant in HA, whereas the mixed-acid fermentation pathway (FERMENTATION-PWY) showed weak but statistically significant enrichment in HU samples.

For ITS-based predictions, metabolic pathways were divided into two groups (Fig. 6E): pathways present in both sample groups and pathways present only in HU samples. ANCOM2 analysis showed that metabolic pathways present in both groups did not differ statistically significantly (Fig. 6F). Six metabolic pathways were more abundant in HU samples, primarily including pathways related to nucleotide synthesis, as well as pathway PWY-7118, which is associated with chitin degradation to ethanol.

To validate the results of metabolic pathway predictions using ANCOM2, we also analyzed the distribution of individual ECs in HU and HA samples, with particular attention to enzymes involved in homofermentative and heterofermentative lactic acid fermentation, alcoholic fermentation, mixed-acid fermentation, acetic acid formation, lactose degradation, and related processes (EC1.1.1.1, EC1.1.1.27, EC4.1.1.1 etc.). The EC analysis revealed the presence of enzymes participating in these pathways; however, no statistically significant differences were detected between samples.

## Discussion

Milk and dairy products constitute a significant proportion of the diet in rural Mongolian populations. During the summer, dairy products can account for 22–48% of total energy intake^55–57^. Notably, molecular analysis of remains from Bronze Age Mongolian burials revealed no evidence of lactase persistence^55^, and lactose intolerance is prevalent in contemporary Mongolic populations (87.9–95% among Mongols, 89% among Buryats)^58–60^. Thus, the processing and fermentation of milk are of critical importance to both the ancient and modern economies of Mongolic peoples.

The analysis of the microbial community of khurenge starter culture showed that it is dominated by thermophilic lactic acid bacteria, primarily *Lactobacillus helveticus*. The prevalence of *L. helveticus* aligns with previous observations of its dominance in Mongolian fermented dairy products such as airag and tarag^24,25^. This may be attributed to the species’ adaptation to the temperature gradient that occurs as milk gradually cools from 37 °C after milking to ambient temperature^61^. *L. helveticus* is able to metabolize lactose, galactose, and glucose through homofermentative and mixed fermentation, producing lactate, acetate, succinate, formate, and ethanol^62^. The presence of *Lactococcus lactis* as a minor component indicates mesophilic fermentation, while detection of *Lactobacillus kefiranofaciens* and *Lentilactobacillus kefiri* is noteworthy since these species are components of kefir grains and have the capacity for exopolysaccharide production^16^.

Among the yeasts present in khurenge, *Trichosporon coremiiforme* and *Monosporozyma unispora* (formerly *Kazachstania unispora*) are dominant. *T. coremiiforme* has previously been isolated from fresh milk and cheese^63^. However, members of this species are incapable of fermentation, though they can utilize lactose, galactose, and glucose during aerobic growth^64^. *M. unispora* is widely found in fermented dairy products^65–67^. *M. unispora* is able to ferment galactose, but not lactose. However, in the presence of lactic acid bacteria, the population of *M. unispora* was found to be higher than the expected population when grown in lactose alone, suggesting that LAB facilitate the breakdown of lactose into glucose and galactose^69^. This may explain why *M. unispora* is considered the primary microorganism responsible for ethanol production in kumis^70^. Thus, it may be hypothesized that ethanol formation in khurenge likewise depends on the functioning of a complex community of yeasts and lactic acid bacteria. Multiple parallel processes likely occur in khurenge, such as lactic acid formation via lactic acid fermentation, alcoholic fermentation by yeasts, and aerobic respiration. This suggests that the traditional technology, which does not employ methods to maintain anaerobiosis, control the precise composition of the microbial community, or optimize the processes, could be improved.

The implementation of modern milk pasteurization standards has a significant impact on the microbial composition of khurenge. Home-produced khurenge (HA), which does not undergo thermal treatment, is characterized by greater microbial diversity, with dominance of non-fermentative *T. coremiiforme* over the ethanol-producing *M. unispora*, lower ethanol concentration (1.5%), and the presence of dissolved β-lactoglobulin in the whey. Industrially produced khurenge (HU) has a narrower microbial composition, dominant yeast *M. unispora*, higher ethanol concentration (2.1%), and absence of dissolved β-lactoglobulin in the whey. From the perspective of discovering new microorganisms with probiotic properties, non-pasteurized fermented dairy product samples may be of particular interest for further study.

The functional metagenomic analysis revealed substantial metabolic complexity in khurenge communities, with 1,473 bacterial and 692 fungal ECs predicting 294 and 50 metabolic pathways, respectively. While overall pathway variance did not differ between HA and HU, composition differed significantly, indicating distinct functional organization patterns despite comparable metabolic heterogeneity. Both samples shared core cellular pathways, yet ANCOM2 identified 107 discriminatory pathways, including enrichment of the hexitol fermentation pathway in HA and mixed-acid fermentation in HU. Notably, individual EC analysis detected no statistically significant differences between samples despite substantial pathway-level differentiation. Both samples showed predicted presence of enzymes for alcoholic fermentation performed by yeasts and lactic acid fermentation. This apparent contradiction is illuminated by the measured ethanol content: HA samples, despite possessing greater metabolic capacity for ethanol fermentation (enriched hexitol pathway), produced lower ethanol (1.5% v/v) compared to HU (2.1% v/v). The inverse relationship between ethanol fermentation pathway enrichment and final ethanol yield suggests that metabolic outcome reflects not ethanol production capacity but rather the balance between competing pathways: HA communities partition available substrate among multiple competing fermentation routes (hexitol, mixed-acid), while HU communities concentrate metabolism into fewer pathways, favoring ethanol accumulation.

The low final ethanol content in fermented khurenge does not allow it to be preserved for extended periods. In this situation distillation is used for long-term preservation of dairy products by peoples of Mongolic origin, predominantly residing in Mongolia, Inner Mongolia (China), Buryatia, and Kalmykia (Russia)^12,13,17^. Production of milk-based distilled alcohol has also been recorded among the Khakas and Tuvans living in the Altai region^18,21^. Notably, distillation of alcohol from milk is unknown among other nomadic Eurasian societies, such as the Yakuts in Siberia and the Kazakhs, despite their advanced traditional dairy processing technologies^13,20^ (Fig. 1). Islam’s prohibitive stance on alcoholic beverages could explain the absence of a contemporary culture of milk-based alcoholic distillates among Kazakhs, who adopted Islam following the collapse of the Golden Horde^71^. In contrast, the predominantly non-Muslim Mongolic peoples may have preserved traditions of celebrations involving alcohol. The adoption of distillation among Eurasian nomads probably occurred during the Mongol Empire, as evidenced by written records and the discovery of distillation apparatuses in archaeological excavations^11^. Travel diaries of visitors to the court of the Mongol Empire also attest to the existence of large-scale alcohol production^8,44^. The existing complex of traditional technologies among the Buryats enables the production of strong alcoholic beverages from milk with the alcohol content up to 50–70% by volume. The use of multistage distillation allows for overcoming the limitation of milk’s low fermentable carbohydrate content, but it substantially increases labor, raw material and fuel consumption, and production costs.

The limited distribution of the technology for producing alcoholic distillates from milk may be related to the low efficiency of the process. Generally, plant substrates contain 1–2 orders of magnitude more fermentable carbohydrates than milk. For example, the glucose yield from wheat starch is 728 kg per ton, theoretically allowing the production of up to 463 L of ethanol per ton of wheat^72^. In contrast, lactose content in cow’s milk is only 4.7%^73^. Based on the stoichiometric equation C₁₂H₂₂O₁₁ + H₂O → 4 C₂H₅OH + 4 CO₂, the theoretical ethanol concentration from complete fermentation of lactose is approximately 3.2% (v/v). Thus, milk is one of the least efficient substrates for ethanol production. It can be hypothesized that the development of ethanol production from milk by distillation occurred under conditions of limited or no stable access to substrates more suitable for alcoholic fermentation.

It is important to note that Mongolian nomads had access to imported grains and could cultivate them when climatic and political conditions were favorable. For instance, isotopic analysis of bone collagen and dental enamel from Early Iron Age burials in Mongolia shows an increase in C4 plant markers, indicating consumption of millet^74^. Reports from Cossack detachments, who contacted the Buryats in 1640–1641, also mention millet cultivation alongside animal husbandry: “the people on that island are mostly Bratskiye (the name for Buryats at the time), with many horses and all kinds of livestock, and they grow millet as grain”^75^. Nevertheless, most historical evidence points to the dominance of pastoralism over agriculture among the Buryats, and dairy products, including khurenge, held great importance in the ritual traditions of Mongolian peoples^76^.

The study of Buryat traditional milk fermentation and distillation technology holds multifaceted scientific, biotechnological, and cultural significance. Globally, distilled alcoholic beverages originate almost exclusively from high-carbohydrate plant substrates, whereas Buryat (and broader Mongolic) systems represent one of very few independently developed “alcohol from milk” technological complexes—a rarity that positions this technology as a unique experiment in how human societies solved ethanol production and preservation under extreme substrate constraints (fermentable sugars 1–2 orders of magnitude lower than in grain-based systems). From a biotechnological perspective, Buryat fermentation provides a valuable complement to industrial high-efficiency processes such as Carbery whey-to-ethanol technology, illustrating two distinct design strategies: centralized, energy-intensive, high-yield lactose fermentation versus decentralized, low-input, equipment-minimal fermentation and distillation adapted to pastoral, resource-limited settings^77,78^. Furthermore, Buryat technology exemplifies circular bioeconomy principles at the small scale: fermentation and distillation co-produce not only alcohol but also dried curd, residual fats, and acidic byproducts, minimizing waste in a pre-industrial context. Finally, as traditional milk spirit production persists today almost exclusively among Mongolic-speaking peoples and a few neighboring Siberian groups, documenting this fermentation technology is crucial for safeguarding intangible cultural heritage, filling a critical gap in global alcohol-technology history, and informing food, tourism, and heritage policy frameworks in Central Asia and Siberia.

## Materials and methods

### Historical and etymological analysis

The historical and etymological study of Buryat and Mongolian terminology was conducted based on written sources, including inscriptions on museum exhibits that preserve references to relevant terms. The analysis employed historical-linguistic approaches, including word-formation and semantic analysis, as well as comparative-historical and etymological methods.

### Description of samples

Two types of khurenge and one type of darasun were investigated. Khurenge sample HA was produced in a household on 30 July 2022 in the Republic of Buryatia, Kurumkansky district, Argada ulus. The sample was obtained on 2 August 2022, with microbial plating performed on 3 August 2022. Khurenge sample HU was produced on 20 July 2022 by the individual entrepreneur Tsydipov in Ulan-Ude, Republic of Buryatia. The sample was obtained on 27 July 2022, and plating was conducted on 28 July 2022. Darasun sample was produced from khurenge in Argada ulus, Kurumkansky district, Republic of Buryatia in summer 2022. The description of the technology was based on interviews with private producers of khurenge and darasun in Argada ulus, as well as on the analysis of literature data.

### Microbiological analysis

Microbial enumeration was performed using the limit dilution method under aerobic conditions with MRS medium supplemented with 20% sterile milk (fat content ≤ 0.5%) for lactic acid bacteria isolation and Sabouraud agar for yeast enumeration. Incubation was carried out at 30 °C.

### Chemical composition

Titratable acidity was determined according to Russian state standard GOST 3624-92 “Milk and Dairy Products—Titrimetric Methods for Acidity Determination”. Experimental distillation of darasun to obtain arza and horzo was performed using a glass distillation apparatus with a Liebig condenser to obtain multiple distillation fractions. Ethanol concentration was measured using gas chromatography.

Carbohydrates and organic acids (except acetic acid) were analyzed by GC-MS after derivatization to trimethylsilyl derivatives^79^. Samples (300 ml) were heated at 70 °C for 30 min, centrifuged (25,000 g, 30 min, 4 °C), and the supernatant filtered through a 0.22 μm PES membrane (Millipore Express PLUS). Whey proteins were removed by filtration using a 5 kDa cutoff membrane (Sartorius Stedim Biotech) and confirmed by Bradford assay. Filtrates (150 ml) were evaporated to dryness, derivatized with hexamethyldisilazane, pyridine, and trimethylchlorosilane, then analyzed by GC-MS using an Agilent 7890 A with a 5975 C detector and HP-5MS column. The column temperature was programmed from 80 to 280 °C at 4 °C/min. Injector and detector temperatures were 250 °C; helium carrier gas flow was 40 cm³/min with a 1:10 split ratio. The mass spectrometer operated in total ion current mode, scanning m/z 50–900. Identification was based on retention times and mass spectra compared to standards.

Acetic acid content was determined using HPLC^80^. Prior to analysis, samples were distilled and fractions collected at 90–100 °C. The clear distillate had a pH of 3.5. HPLC analysis was performed using an Agilent 1200 Series system equipped with a diode array detector (DAD) G1315C, binary dual pump G1312B, autosampler G1367C, degasser, and column thermostat (Agilent Technologies, USA) controlled by Agilent ChemStation software (ver. B 04.03 SP1). A reversed-phase PerfectSil 300 ODS C18 column (5 μm, 4.6 × 250 mm, MZAnalysentechik, Germany) was used at 25 ± 1 °C. Detection wavelength was 210 nm; flow rate was 0.5 ml/min; mobile phase was 0.01 M phosphate-buffered saline (PBS) adjusted to pH 2.5.

Total protein concentration was measured in both whole khurenge samples and whey. Whey was prepared by centrifugation at 30,000 g for 30 min, followed by filtration through a 0.20 μm PES membrane. Samples were mixed 1:1 with 20 mM Tris pH 7.8, 6 M guanidine chloride buffer. Protein quantification was performed using the bicinchoninic acid (BCA) assay (Sigma-Aldrich, St. Louis, MO, USA), with BSA (1 mg/ml) as a standard. Measurements were performed at various dilutions and in duplicate, following microplate protocol—mixing 25 µl sample with 200 µl working reagent, incubation at 37 °C for 30 min, and absorbance reading at 562 nm. Protein concentration was determined using a calibration curve generated for each experiment.

SDS-PAGE was performed using Laemmli’s method^81^ in 12% and 15% polyacrylamide gels (Mini-PROTEAN Tetra Cell system, Bio-Rad, Hercules, CA, USA). Samples were heated with sample buffer at 95 °C for 30 min, loaded, and electrophoresed at 120 V (stacking gel) and 180 V (separating gel). Protein bands were visualized using Coomassie-R250 stain. Molecular weights were evaluated with a pre-stained marker (Thermo Scientific, Waltham, MA, USA). Whey samples were prepared by centrifugation; precipitates were suspended in 40 mM Tris pH 7.8, 40 mM NaCl.

### Sequencing of DNA

DNA was extracted using Qiagen PowerLyzer PowerSoil kit (Qiagen, Hilden, Germany) according to manufacturer’s instructions. The amplicon libraries of the hypervariable V4 region of the 16 S rRNA gene were prepared using the two-stage PCR strategy, as described previously^82^. Briefly, approximately 2 ng of DNA and two negative controls were used for the first round of amplification with fusion primers, containing partial TruSeq adapter, heterogeneity spacer^83^ and rRNA primers 515F^84^ and Pro-mod-805R^85^. Amplification was performed by CFX96 Touch Real-Time PCR Detection System (Bio-Rad, Hercules, CA, USA) with the parameters described previously^82^. Amplificated mix, diluted 2–5 times (depending on Ct) was used as a matrix for the second PCR with double index-containing primers^86^. Each PCR was performed in two replicates, resulting in 34 V4 amplicon libraries. Libraries were checked with agarose gel and pooled in equimolar amounts. The final pool was cleaned with AMPure XP beads (Beckman Coulter, Brea, CA, USA), according to manufacturer’s instructions. Libraries were sequenced with the MiSeq™ Personal Sequencing System (Illumina, San Diego, CA, USA) using the 156-bp paired-end reads.

### Processing of ITS and 16 S rRNA gene amplicon-sequencing data

Sequencing data were processed in R (version 4.4.2). The Divisive Amplicon Denoising Algorithm 2 (DADA2)^87^ pipeline (dada2 package version 1.34.0) was used for data processing (see script ‘KhurengeDada2.Rmd’ on GitHub https://github.com/andwhoami/khurenge). All functions were run with recommended parameters (https://benjjneb.github.io/dada2/tutorial.html*)*, except for the “expected errors” parameter, set to maxEE = 0.5 in the filterAndTrim function. Taxonomic assignments were made using the SILVA^88^ database V138.1 for bacteria and the UNITE database version 8.3 (2021-05-10) for fungi^89^. Reads mapped to mitochondria and chloroplasts were excluded using the subset_taxa function from the phyloseq package (version 1.28.0)^90^. Species present in less than 1% abundance in only one sample were excluded using the core_members function (microbiome package version 1.28.0, http://bioconductor.org/packages/microbiome/). Species found in more than two samples but with less than 5% prevalence in each were grouped as “Other”.

### Diversity analysis and statistics

Beta-diversity distances and Principal Coordinates Analysis (PCoA) visualisations were calculated using the dist function from the stats package (version 4.4.2) for bacteria and fungi separately. Multivariate homogeneity of group variances was assessed using the betadisper function, and partitioning of distance matrices among sources of variation with linear model fitting was performed using the adonis2 function (vegan package version 2.7-1; see part ‘Beta-diversity’ in script ‘KhurengeAnalysis.Rmd’ on GitHub https://github.com/andwhoami/khurenge)(Dixon, 2003). Classical multidimensional scaling (MDS) for PCoA visualisation was performed using the cmdscale function (stats package). Visualisations were generated with the geom_point function from the ggplot2 package (version 3.5.2)^91^. Alpha diversity indices (Simpson, Shannon, Chao1) were computed using the vegan package. Comparisons were performed using the Wilcoxon test, with p values adjusted using the Bonferroni method. All chemical analyses and microbiological analysis were performed in triplicate. For microbial diversity analysis two samples from each khurenge type were analyzed, each in two independent PCR replicates.

### Microorganism composition and cluster analysis

Relative abundances were used to describe microorganism composition. For visualisation, microbial taxa were grouped at the genus level and log-transformed (log10) separately for bacteria and fungi. Plots were generated using the pheatmap package (version 1.0.13, https://github.com/raivokolde/pheatmap). Sample and genus clustering in the heatmaps utilised Euclidean distances on log10-transformed data (cluster_rows and cluster_cols parameters.

### Metagenomic functional prediction and pathway analysis

We used PICRUSt2 (Phylogenetic Investigation of Communities by Reconstruction of Unobserved States) version 2.4.1 to infer metagenomic functional profiles from 16 S rRNA and ITS gene amplicon data^92^. Amplicon sequence variant (ASV) tables and corresponding representative sequences were provided to the picrust2_pipeline.py script with default parameters. Nearest Sequenced Taxon Index (NSTI) values were examined to ensure acceptable phylogenetic placement for 16 S data. For ITS data, the PICRUSt2 fungal database was used. The resulting predicted gene family and MetaCyc pathway abundance tables were used for downstream statistical analyses in R.

For initial analysis of differences in metabolic pathways, betadisper with permutations was applied to assess variance differences between the HA and HU groups, and adonis2 was used to evaluate dissimilarity in metabolic pathway profiles. Principal coordinate analysis (PCoA) was then performed, and the resulting ordinations were visualized using the ggplot2 package. To identify metabolic pathways enriched in each sample group, log₁₀-transformed pathway abundances were visualized using the pheatmap package, with both samples and pathways clustered based on Euclidean distances. Finally, to detect sample-specific metabolic pathways, ANCOM2 was applied, and statistically significant pathways were visualized as bar plots using ggplot2 (geom_bar) ^93^.

## Supplementary Information

Below is the link to the electronic supplementary material.

Supplementary Material 1

Supplementary Material 2

## Data Availability

Sequencing data generated in this study have been deposited in the NCBI Sequence Read Archive (SRA) under accession number PRJNA1307465.
