# Supplementary material for: Microbial diversity and production of milk spirit using traditional Buryat fermentation and distillation technologies
Source: Sci Rep. 2026 Apr 16;16:17769. doi: 10.1038/s41598-026-45709-5 (PMC13247081; doi:10.1038/s41598-026-45709-5)

Supplementary materials

Microbial diversity and production of milk spirit using traditional Buryat fermentation and distillation technologies

Zorigto Namsaraev[1,2,3], Bair Nanzatov[4], Darima Barkhutova[5], Aleksandra Kozlova[6,7], Anna Izotova[1], Anna Vlaskina[1], Dmitry Petrenko[1,3], Andrey Kamaev[1], Svetlana Lukashevich[1], Viktor Pozhidaev[1], Alla Filimonova[1], Dulma Tsyrenova[5], Vyacheslav Dambaev[5], Valeriia Novikova[2], Aleksei Rozanov[2], Alexey Sazonov[2], Maksim Patrushev[1], Stepan Toshchakov[1]

[1]NRC “Kurchatov Institute”, Moscow, Russia

[2]Sirius University of Science and Technology, Sirius Federal Territory, Russia
[3]Moscow Center for Advanced Studies, Moscow, Russia

[4]Institute for Mongolian, Buddhist and Tibetan Studies, Siberian Branch of the Russian Academy of Sciences, Ulan-Ude, Russia

[5]Institute of General and Experimental Biology, Siberian Branch of the Russian Academy of Sciences, Ulan-Ude, Russia

[6]International Laboratory of Bioinformatics, HSE University, Moscow, Russia
[7]Center for Molecular and Cellular Biology, Moscow, Russia

Supplementary figure 1: Rarefaction curve based on ITS sequencing data

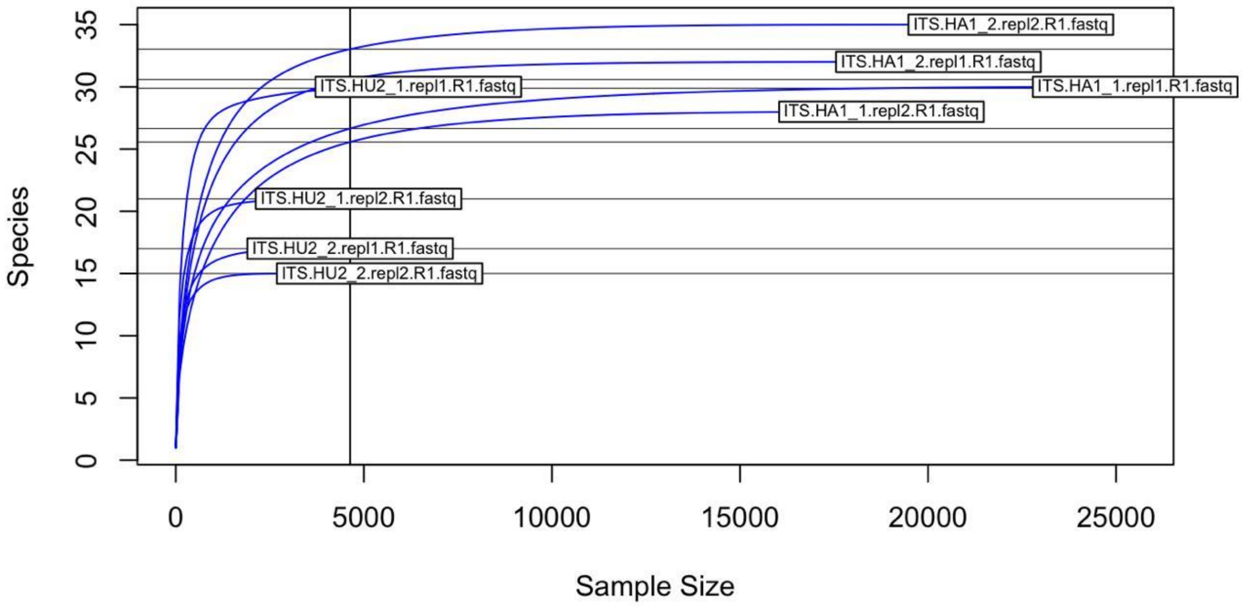

Supplementary figure 2: Rarefaction curve based on 16S rRNA gene sequencing data

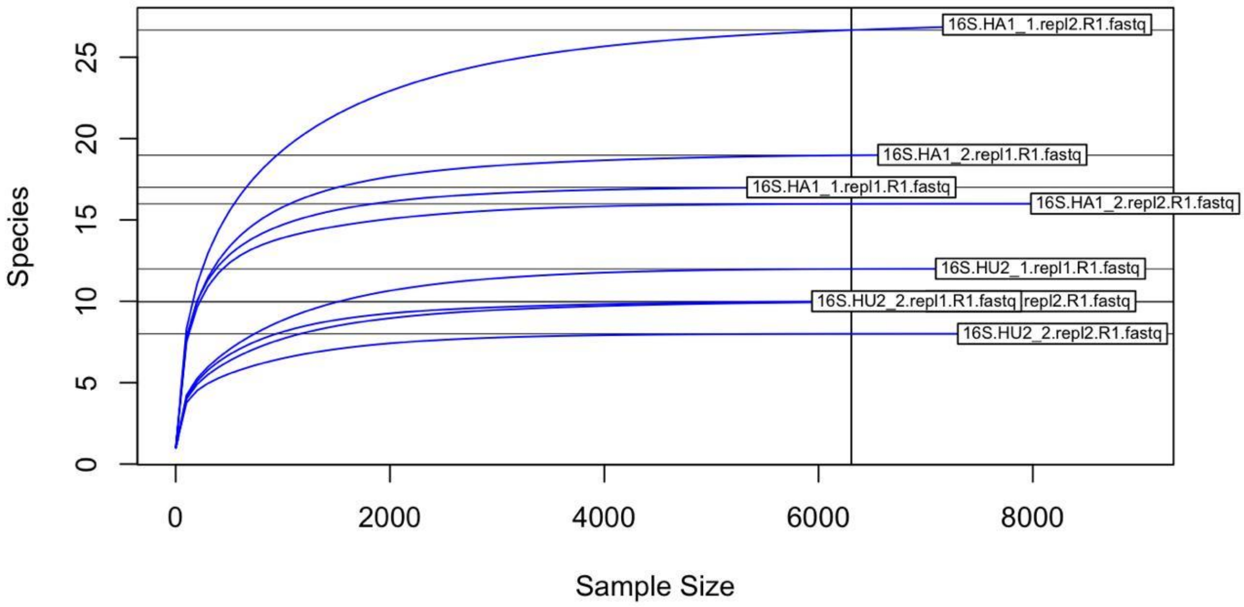

Supplement: Supplementary file 1 — Supplementary Material 1 [file 41598_2026_45709_MOESM1_ESM.pdf]
